# Esotropia Missed During Pre-health Checkup Screening With the Spot Vision Screener: A Case Series

**DOI:** 10.7759/cureus.104609

**Published:** 2026-03-03

**Authors:** Keiko Fuseya, Tadashi Matsumoto, Saiko Matsumura, Momoko Kawakami, Erika Chiba, Masahiko Tomita, Yoko Kunugi, Yuichi Hori

**Affiliations:** 1 Department of Ophthalmology, Toho University Faculty of Medicine, Tokyo, JPN

**Keywords:** accommodative esotropia, amblyopia, false-negative results, pediatric vision screening, spot vision screener (svs)

## Abstract

The Spot Vision Screener (SVS) is increasingly used for the early detection of amblyopia risk factors in preschool children, particularly during health checkups for three-year-olds. Although SVS demonstrates high sensitivity and specificity for refractive errors and amblyopia risk factors, its ability to detect strabismus, especially accommodative esotropia, remains limited. We present a case of accommodative esotropia in children under three years of age where SVS failed to detect ocular misalignment. In all cases, SVS indicated only mild hyperopia and did not detect strabismus; however, subsequent ophthalmological assessment revealed accommodative esotropia, which was confirmed by cycloplegic refraction. All patients responded well to full hyperopic correction with spectacles, which resulted in improved or normalized ocular alignment. These cases highlight the potential for false-negative strabismus results with SVS, particularly in young children and in cases where esotropia is variable or predominantly manifests at near. Our findings underscore the need for a comprehensive clinical assessment, including careful observation, cycloplegic refraction, and repeated SVS measurements, to ensure timely and accurate referral for ophthalmological evaluation. Enhanced education and training for healthcare professionals involved in vision screening are also essential to maximize the detection rates of amblyopia and strabismus and to prevent missed diagnoses of clinically significant strabismus.

## Introduction

Early detection and intervention for amblyopia and its risk factors are critical in pediatric ophthalmology, as delayed diagnosis can result in irreversible visual impairment. Amblyopia is the leading cause of monocular vision impairment in children, and early treatment within the critical developmental period is essential for optimal outcomes [1,2]. Instrument-based vision screeners, such as the Spot Vision Screener (SVS; Welch Allyn, New York, US), have been incorporated into Japan's three-year-old health examination system, with studies showing high measurement success rates of 99.8% and effective detection of amblyopia risk factors [3,4]. The SVS is a handheld photoscreening device that enables rapid, non-invasive assessment of refractive errors, anisometropia, and ocular alignment at a distance of approximately 1 meter [5]. This device utilizes infrared photorefraction technology to simultaneously measure both eyes within seconds, providing objective information on refractive status, pupil size, and ocular alignment [6].

According to validation studies, the SVS demonstrates high sensitivity for detecting amblyopia risk factors, with Garry and Donahue reporting sensitivity of 85-89% and specificity of 71-88% depending on the referral criteria used [7]. Peterseim et al. demonstrated that the SVS achieved a sensitivity of 87.7% and a specificity of 75.9% for detecting amblyopia risk factors [5]. These studies indicate that the SVS is a valuable tool for large-scale screening initiatives due to its consistent performance across different populations and settings. While the SVS has been validated for refractive error detection, its performance in identifying strabismus, especially accommodative esotropia, is less robust [8]. Studies indicate that the sensitivity for strabismus detection varies significantly depending on the study conditions and population examined [6]. The detection of strabismus remains challenging for photoscreening devices, as small-angle strabismus or intermittent deviations may not be consistently detected [9].

In Japan, the use of SVS is expanding not only in three-year-old health checkups but also in pediatric and ophthalmology clinics [4]. Japanese studies have shown that incorporating SVS into regular health checkups significantly improves the detection rate of undiagnosed amblyopia, especially monocular amblyopia with binocular dysfunction, which tends to be overlooked in conventional visual acuity tests performed at home [4]. However, there is limited literature on strabismus detected by SVS, and to our knowledge, there are almost no reports on children under three years of age. In this report, we present three cases of accommodative esotropia in which only mild hyperopia was noted on SVS performed prior to the three-year-old health checkup, and no ocular misalignment was detected. This case report emphasizes that while the SVS examination is a simple and highly sensitive test, considering the presence of some false-negative cases, it is important to continue using traditional methods, such as interviews and visual examinations, in addition to the SVS examination.

## Case presentation

Case 1

**Figure 1:**
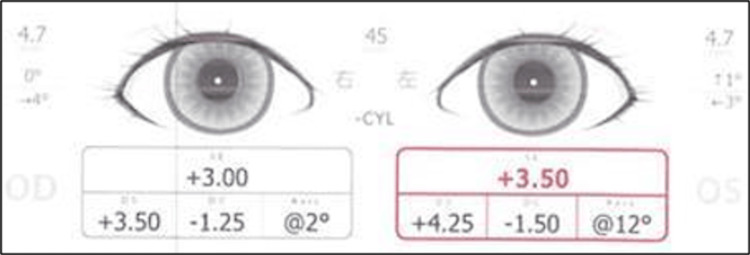
SVS findings at the initial examination (Case 1) Eye position was within the normal range, but mild hyperopic astigmatism was observed. SVS: Spot Vision Screener

This involved a 1-year and 8-month-old boy who was referred to our hospital for suspected abnormal ocular alignment, despite a normal SVS result at his 18-month health checkup. At presentation, SVS detected only mild hyperopia, and ocular alignment was reported as normal (Figure 1).

However, clinical examination revealed esotropia at both near and distance fixation. Cycloplegic autorefraction (Retinomax, Righton, Japan) showed +4.50 D hyperopia in both eyes. The diagnosis of accommodative esotropia was made, and full hyperopic correction was prescribed. After three months, ocular alignment improved under spectacle correction, although some residual esotropia persisted at near. The causes of residual esotropia considered included partial accommodative esotropia or the possibility that insufficient eye drops due to atropine side effects resulted in incomplete cycloplegia.

Case 2

**Figure 2:**
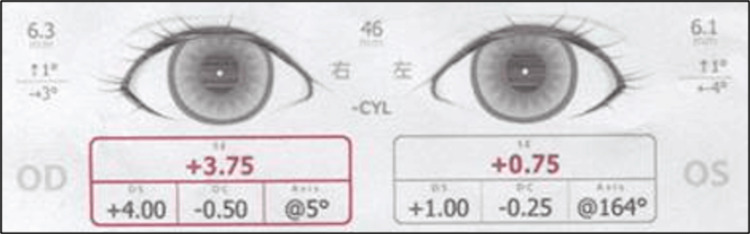
SVS findings at initial examination (Case 2) Eye position was within the normal range, but hyperopia was observed. SVS: Spot Vision Screener

This was a 1-year and 11-month-old girl referred for further evaluation after SVS screening at a nursery school suggested anisometropia, but no strabismus. At our clinic, SVS again showed hyperopia without misalignment (Figure 2).

**Figure 3:**
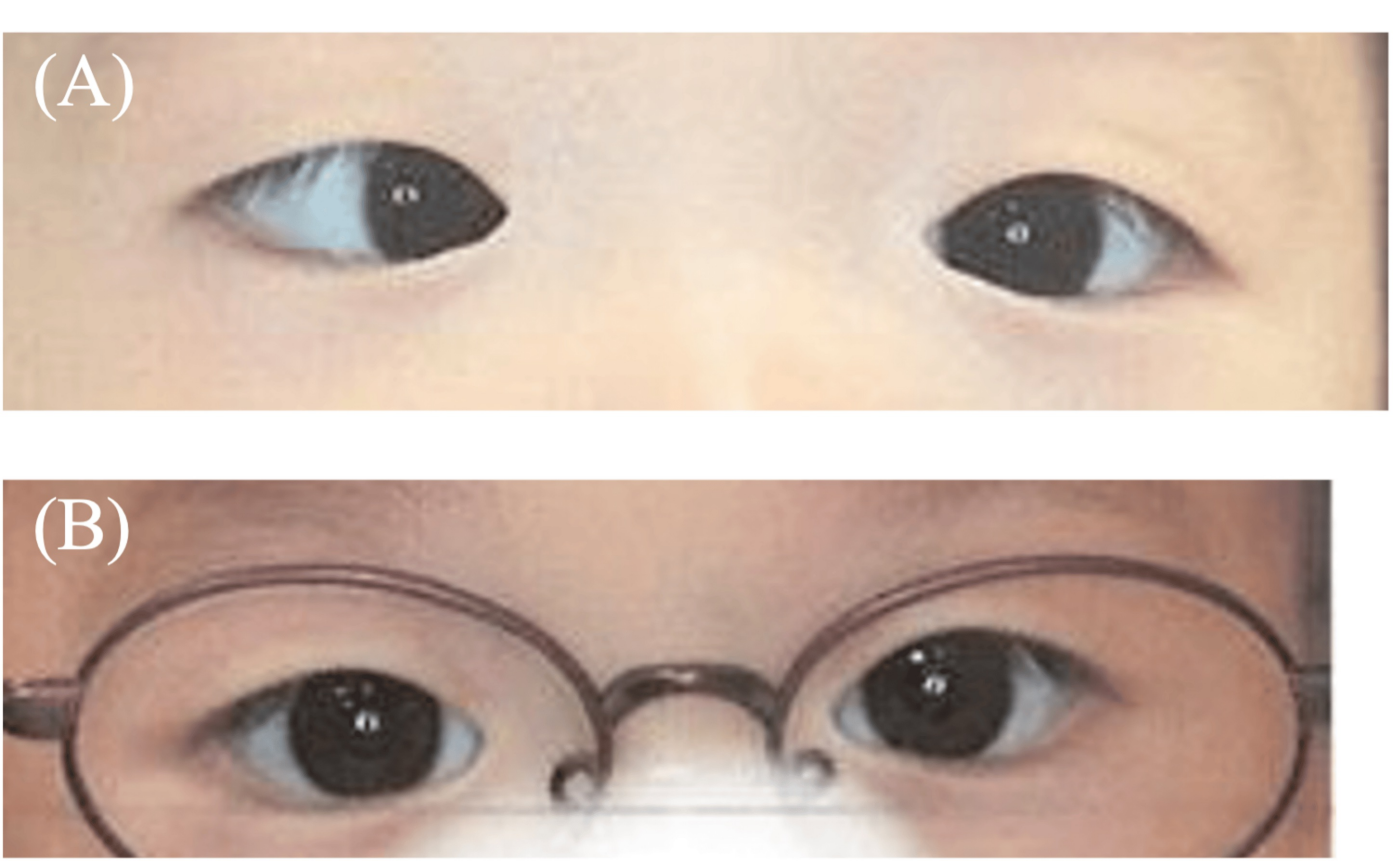
Photographs of eye position at the initial examination (A) and after three months following prescription of fully corrected glasses (B) (Case 2) In (A), right esotropia was observed, and in (B), it can be confirmed that the eye position was normal.

On examination, esotropia was evident at near fixation, with orthotropia at distance. Cycloplegic autorefraction (Retinomax) revealed +4.75 D in the right eye and +2.50 D in the left. With full correction glasses for both eyes, the eye position was normalized (Figure 3).

**Figure 4:**
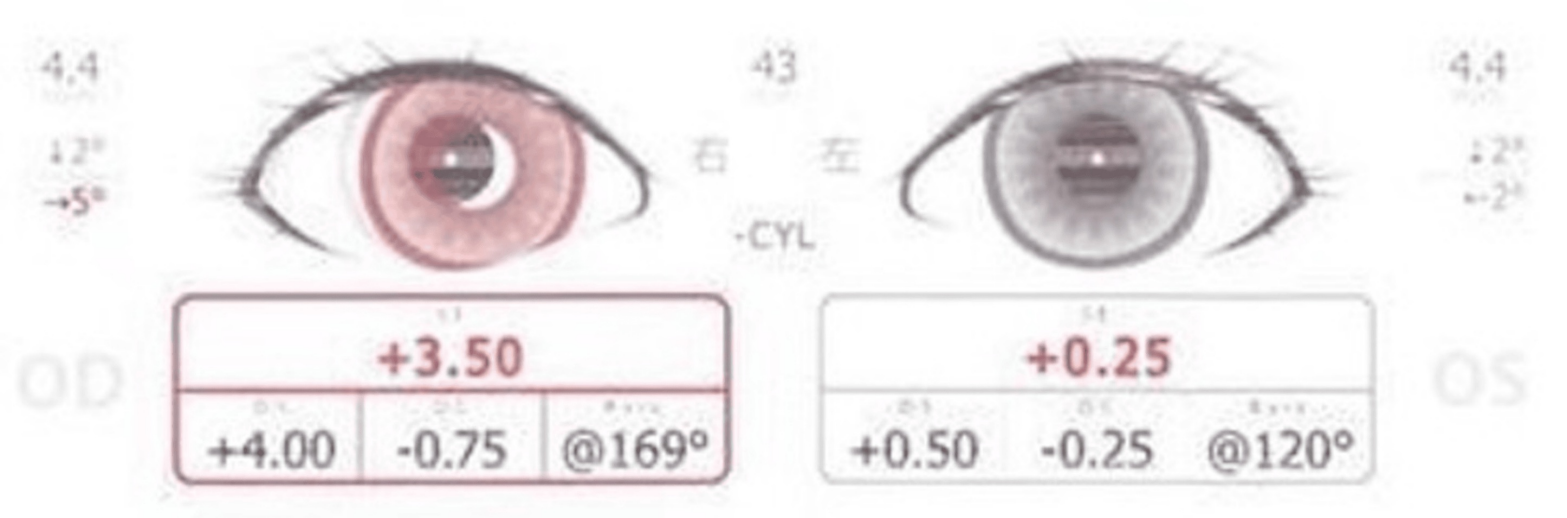
SVS findings under observation (three years old) (Case 2) Right eye esotropia was observed, a reduction in hyperopia was observed, as well as mild miosis of the pupil. SVS: Spot Vision Screener

Notably, measuring SVS at age three detected esotropia and showed mild miosis with reduced hyperopia (Figure 4).

Case 3

**Figure 5:**
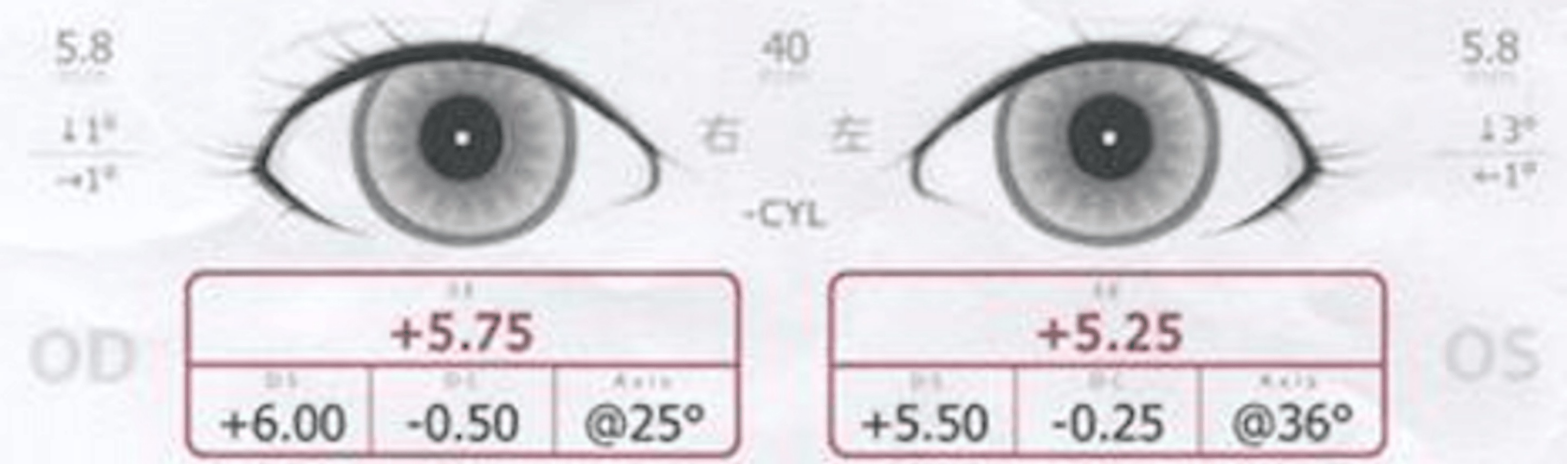
SVS findings at initial examination (Case 3) Eye position was within the normal range, but hyperopia was observed. SVS: Spot Vision Screener

This involved a 1-year and 2-month-old girl with a history of myotonic dystrophy, referred after SVS screening in a pediatric clinic, which indicated that she had hyperopia but no strabismus. At our hospital, SVS again showed only hyperopia (Figure 5).

**Figure 6:**
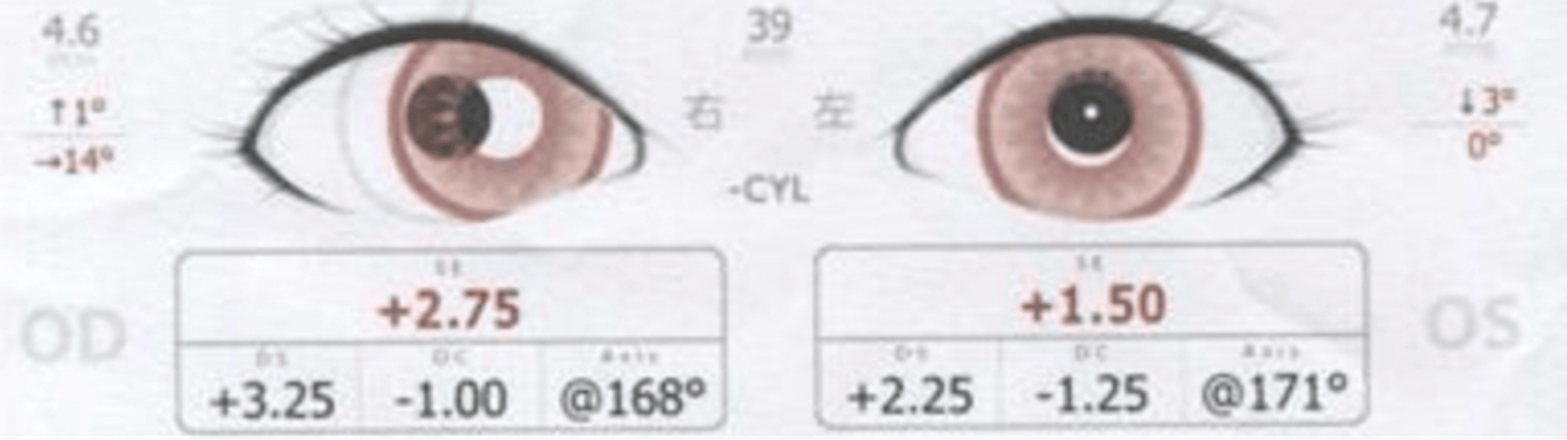
SVS findings under observation (at 1 year and 6 months) (Case 3) The eye position showed esotropia in the right eye, refraction showed a reduction in hyperopia, and mild miosis of the pupil was observed. SVS: Spot Vision Screener

Examination revealed esotropia at both near and distance. Cycloplegic autorefraction (Retinomax) showed +5.50 D hyperopia in both eyes. With full corrective glasses for both eyes, the eye position was normalized. Repeat SVS at 1 year and 6 months identified esotropia and mild miosis, with reduced hyperopia (Figure 6).

## Discussion

Our case series highlights critical limitations of the SVS in detecting accommodative esotropia, particularly in children under three years of age. While previous studies have demonstrated that the SVS achieves high sensitivity (85-89%) and specificity (71-88%) for detecting amblyopia risk factors in general populations [5,7], our findings reveal significant false-negative results for accommodative esotropia associated with moderate hyperopia. This aligns with recent reports suggesting that photoscreening devices may have reduced sensitivity when accommodative demand is limited, as the SVS measures refractive error at a distance of approximately 1 meter without providing sufficient accommodative stimulus to unmask latent esotropia [8]. The American Academy of Pediatrics has acknowledged that instrument-based screening methods have inherent limitations, particularly noting that "there is no mechanical substitute at present for an adequate physical examination" [10]. Our cases demonstrate that even with hyperopia of +2.50 to +5.50 diopters, the SVS failed to detect ocular misalignment in all three patients, suggesting that the device's algorithms may not adequately account for accommodative esotropia in very young children.

The clinical implications of these false-negative results are substantial, as accommodative esotropia represents a treatable condition that can lead to amblyopia if not detected and managed appropriately [1]. Early recognition and treatment are important to prevent vision loss, as children younger than seven years are more likely to have a good response to treatment [1]. Previous validation studies have predominantly focused on older preschool children, with limited data available for children under three years of age. Our cases suggest that the SVS may be less reliable in detecting strabismus in younger children, possibly due to developmental differences in accommodative response and the variable nature of accommodative esotropia in this age group. Notably, repeat SVS testing at older ages (around three years) in two of our cases successfully detected the esotropia, indicating that the device's performance may improve with age. This finding underscores the importance of age-specific considerations when interpreting SVS results and the need for continued surveillance even after normal screening results in very young children.

Given these limitations, our findings support the recommendation that SVS screening should be used as a complementary tool rather than a replacement for traditional clinical assessment methods. The combination of instrument-based screening with clinical examination, including careful observation for ocular misalignment and detailed parental history, appears essential for comprehensive vision screening. As noted in current guidelines, "combining two screening tests does not necessarily result in the highest sensitivity and specificity from each component test," but may provide valuable supplementary information [11]. The high measurement success rate and objective nature of the SVS make it a valuable screening tool, but clinicians and screening personnel must remain aware of its limitations, particularly in detecting accommodative esotropia in young children. This case series emphasizes the importance of maintaining traditional screening methods, including parental interviews and clinical observation, alongside instrument-based screening to ensure comprehensive detection of vision disorders in pediatric populations.

## Conclusions

In summary, while the Spot Vision Screener is a convenient and generally sensitive tool for detecting refractive errors and amblyopia risk factors in preschool children, it may fail to detect certain cases of accommodative esotropia, particularly in younger children and when the deviation is variable or primarily evident at near. Our cases demonstrate that reliance on SVS alone can result in false-negative findings for strabismus. Comprehensive clinical evaluation, including careful observation, cycloplegic refraction, and, when necessary, repeated screening, is essential for accurate diagnosis. Education and awareness among healthcare providers are critical to ensure that children with subtle or variable strabismus are appropriately referred for ophthalmological assessment, thereby preventing delayed diagnosis and optimizing visual outcomes.
